# Blood pressure predictors of stroke in rural Chinese dwellers with hypertension: a large-scale prospective cohort study

**DOI:** 10.1186/s12872-019-1186-0

**Published:** 2019-08-29

**Authors:** Jia Zheng, Zhaoqing Sun, Xiaofan Guo, Yanxia Xie, Yingxian Sun, Liqiang Zheng

**Affiliations:** 1 Department of Clinical Epidemiology, Library, Department of Health Policy and Hospital Management, Shengjing Hospital of China Medical University, Shenyang, 110004 People’s Republic of China; 2 Department of Cardiology, Shengjing Hospital of China Medical University, Shenyang, 110004 People’s Republic of China; 3 Department of Cardiology, the First Affiliated Hospital of China Medical University, Shenyang, 110001 People’s Republic of China

**Keywords:** Blood pressure, Stroke, Hypertension

## Abstract

**Background:**

Little was known about the different predictive power of blood pressure (BP) parameters (SBP, systolic BP; mean arterial pressure, MAP; pulse pressure, PP; and diastolic BP, DBP) and stroke incidence. This study’s aim was to compare power of BP parameters predict stroke events among rural dwelling Chinese individuals with hypertension.

**Method:**

A total of 5097 hypertension patients (56.2% women; mean age, 56.3 ± 11.2 years) were included in the prospective cohort study with a median follow-up of 8.4 years.

**Results:**

Until the end of the last follow-up, there were 501 onset strokes (310 ischemic, 186 hemorrhagic, and 5 unclassified strokes) among the 5097 participants. The results showed that hazard ratio (HR) (95% confidence interval, 95% CI) with an increment of 5 mmHg were 1.095 (1.070–1.121) for PP, 1.173 (1.139–1.208) for MAP, 1.109(1.089–1.130) for SBP, 1.143(1.104–1.185) for DBP. The SBP indicated the largest *β* coefficient in the Cox proportional hazard model for all stroke except PP or MAP, and the SBP revealed slightly higher value than MAP (*β*_SBP_ = 0.435, *β*_MAP_ = 0.430, *P* = 0.756).

**Conclusions:**

Both PP and MAP were predictive factors for stroke. The MAP showed a stronger ability to predict stroke events than PP, and slightly inferior to SBP for hypertension patients.

## Background

Stroke is considered to be the second primary cause of death in the world [1], and China has the highest burden of stroke in the glob [2]. In addition, hypertension is considered to be the mainly risk factor of disability-adjusted life-years and deaths [3]. Previous study has indicated that over one-quarter of adult population were hypertension patients in China [4]. For hypertension patients, the incidence of stroke is higher than it in persons with normal BP [5].

Recently, MAP and PP have been used as predictors for stroke events and confirmed in many studies [6–21]. MAP is another measure of the overall circulating pressure load and considered to predict adverse cardiovascular outcomes. The value of MAP can be directly determined by cardiac catheterization or estimated by a formula [e.g., diastolic + 1/3 x (systolic-diastolic)]. However, as the age of the patient increases, the discrimination ability of the mean arterial pressure decreases. DBP peaked at the age of 55 and then decreased, while the systolic blood pressure (SBP) continued to grow with age, and higher every 10 years [22]. Thus, based on the individual changes in SBP and DBP with age, actual changes in MAP tend to be low with age. At the same time, there is increasing emphasis on SBP as the effective indicator of cardiovascular disease (CVD) apart from other BP parameters for the middle-aged and elderly person. The correlation between PP and SBP is more closely related to DBP with an increasing age. The PP is regarded as a proper predictor of CVD in the general population. Nevertheless, few researches have been conducted among hypertensive patients, especially in Chinese [6–9, 23]. It is particularly crucial to discover a strong predictive indicator of stroke in this group, since there were some limitations in previous studies, such as short follow-up time [23], risk factors for stroke adjusted inadequately, or lack of comparisons of HR values among the four BP parameters (PP, DBP, MAP, and SBP) [6–10].

The goal of our study was to explored the relationship between infrequent BP parameters (PP and MAP) and stroke events [All stroke, ischemic stroke (IS), hemorrhage stroke (HS)] in prospective hypertension cohort and compare the predictive values of four BP parameters for stroke events.

## Methods

### Study population

The China Medical University Ethics Committee had approved the study plan and received the written informed consent from all patients or their guardians.

**Fig. 1:**
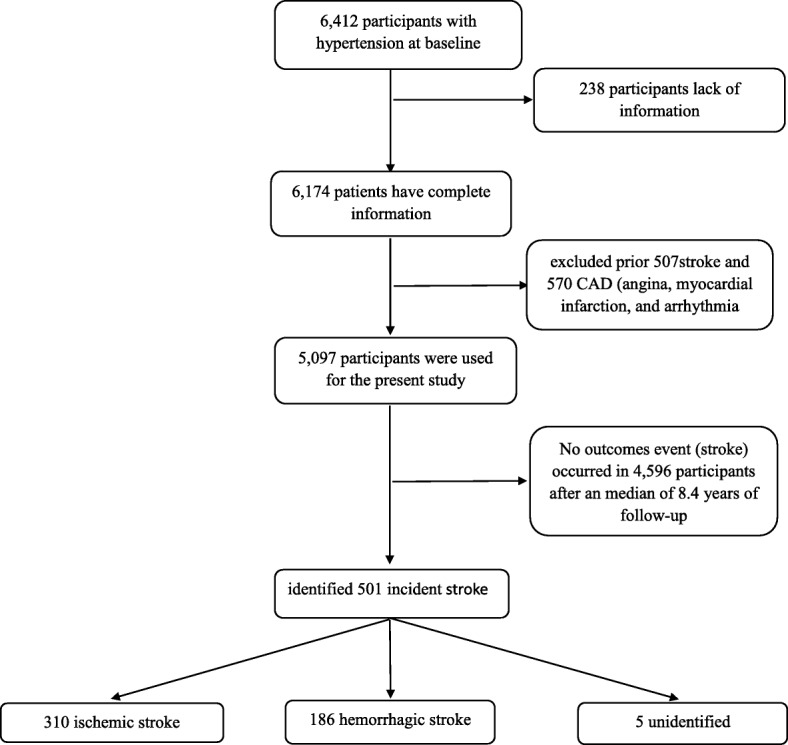
Flow chart of participants included in this study after inclusion and exclusion

Our data comes from a prospective cohort from Fuxin county hypertensive population. The first multistage cluster random sampling design was conducted in 64 rural communities in Fuxin county (including seven cities in five locations: east, west, south, north and central) of Liaoning province, China. Hypertensive patients aged ≥35 years who agreed to take part in the study and signed informed consent forms were included. At the same time, pregnant women, those having malignant tumors, severe hepatic and renal insufficiency, those unwilling to participate in the study were excluded. 6412 hypertensive patients were covered at the start of our study [24, 25]. All the eligible participants were invited to the following visits. And the three follow-up visits as detailed previously [24]. The detailed inclusion process of participants is shown in Fig. 1.

### Study outcomes

Our study endpoint was stroke, according to the MONICA criteria to confirmed the events. The event with the major events in the etiology of blood vessels, including local or global brain disease lasting for more than 24 h, and stroke events due to death or surgical duration less than 24 h [25]. The project diagnostic team confirmed the new stroke events during follow-up visits and the final verification approved. Data classification and measurement on stroke as detailed previously [24].

### BP measurement

Standardized measurement of BP values have been detailed in previous articles [24]. We defined hypertension as the take of anti-hypertensive medications in the last 2 weeks or DBP ≥90 mmHg or SBP ≥140 mmHg. Further calculations of BP parameters were included such as: *PP* = *SBP* − *DBP*; *MAP* = (2 × *DBP* + *SBP*)/3.

### Other risk factors

Information on other risk factors data (serum glucose, lipids, cholesterol) collection and measurement had been described in previously literature [23, 24, 26]. Professional physicians performed face-to-face interviews with patients to obtain lifestyle factors (smoking, drinking, and BP medication). The smoking and drinking were defined as detailed previously [11, 24]. We defined diabetes as current treatment with oral hypoglycemic agents or insulin or fasting serum glucose levels ≥7.0 mmol/l .

### Statistical analysis

The Pearson correlation was applied to assess the correlation coefficient among BP parameters. The Cox proportional hazard model was applied to count the HR value and 95% CI of four BP parameters for the risk of incident stroke. First, four BP parameters in form of continuous variables in the model, and the HRs were calculated with 1 SD mmHg intervals to estimate the relationships among four BP parameters and onset stroke events. The incident stroke was examined to determine the association with four BP values across the age <  60 years and the age of ≥60 years. Multivariable model was adjusted by sex, ethnicity, age, body mass index (BMI), smoking, diabetes mellitus (DM), drinking, heart rate (HR), TC, LDL-C, TG, HDL-C, and anti-hypertensive medications. Principle of comparing the difference in HR values were described in the previous published literature [24]. We used SPSS 22.0 (IBM Inc., Chicago, IL, USA) to conducted data analysis and *P* < 0.05 was regarded to be significant.

## Results

**Fig. 2:**
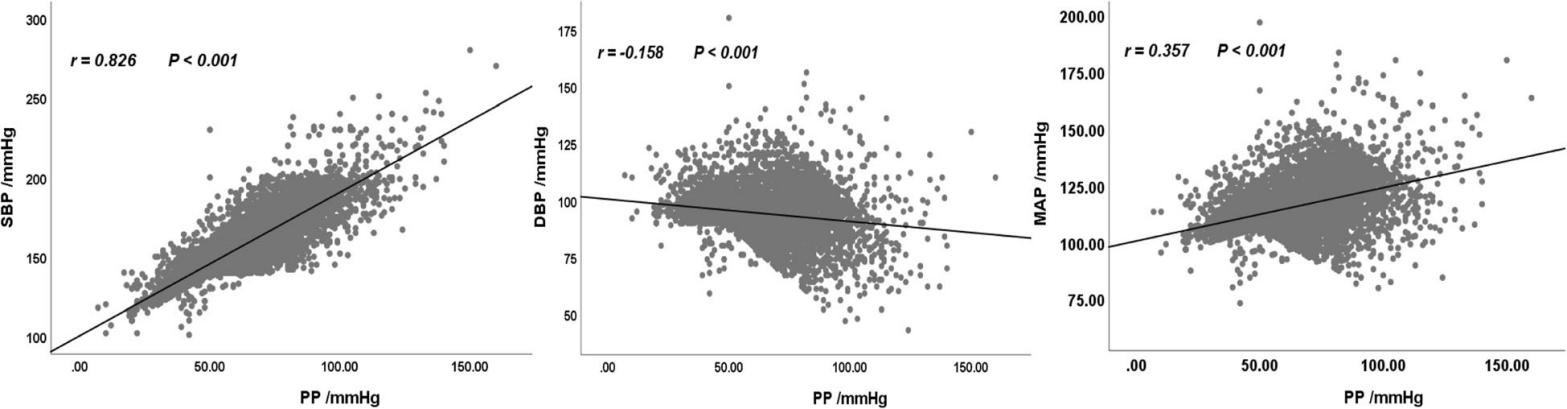
Scatter plot among the blood pressure parameters

Baseline information in this study were showed in Table 1. Baseline mean (SD) age of women was 56.3 (11.2) years of the 5097 initially no-stroke hypertensive patients. A positive correlation between PP and SBP (r = 0.826; *P* < 0.001) was found, but PP revealed negative correlations with DBP (r = − 0.158; *P* < 0.001) and MAP (*r* = 0.357; *P* < 0.001) as can be shown in Fig. 2 .

**Table 1:** Baseline Characteristics of hypertensive patients

Variables	strokes	non-strokes	*P*-value
n	4596	501	
Age, years, Mean (SD)	55.8(11.18)	60.4(10.99)	
Women, n (%)	2653(57.7)	213(42.5)	< 0.001
Han ethnicity, n (%)	3691(80.3)	386(77.0)	0.835
BMI, kg/m2,Mean (SD)	23.9(3.41)	23.5(3.25)	0.011
Current smoking, n (%)	1802(39.2)	243(48.5)	< 0.001
Current drinking, n (%)	1334(29.0)	169(33.7)	0.028
Taking anti-hypertensive drugs, n (%)	1004(21.8)	187(37.3)	< 0.001
HR, beats/min, Mean (SD)	76.0(11.10)	75.6(11.32)	0.505
Diabetes mellitus, n (%)	349(7.6)	52(10.4)	0.028
Lipid,mmol/L, Median (IQR)			
TC	5.16(4.52–5.82)	5.33(4.61–6.01)	0.002
TG	1.31(0.93–1.97)	1.38(0.98–2.09)	0.091
HDL-C	1.40(1.20–1.62)	1.42(1.20–1.64)	0.543
LDL-C	2.72(2.27–3.20)	2.88(2.37–3.34)	0.001
BP Parameters, mmHg, Mean (SD)			
SBP	158.4(20.51)	172.0(21.77)	< 0.001
DBP	93.6(11.69)	97.5(14.00)	< 0.001
MAP	136.8(15.65)	147.2(17.47)	< 0.001
PP	64.8(19.11)	74.5(18.49)	< 0.001

The body mass index (BMI) is the weight in kilograms divided by the square of the height in meters. *BP* blood pressure, *HR* heart rate, *BMI* body mass index, *IQR* inter-quartile range, *TC* Total cholesterol, *TG* Triglycerides, *HDL-C* high-density lipoprotein cholesterol, *LDL-C* low-density lipoprotein cholesterol, *SBP* systolic blood pressure, *DBP* diastolic blood pressure, *PP* pulse pressure, *MAP* mean arterial pressure

501 onset outcomes occurred until the last follow-up visit(310 IS events, 186 HS events, and 5 unclassified stroke events). The incidence density of all strokes were 1236.30 per 100,000 person-years (95% CI: 1167.81–1304.79), IS was 764.97 (95% CI: 710.97–818.97), and HS was 458.99 (95% CI: 417.10–500.89). HRs (95% CI) of future stroke after multivariate adjustment for each BP parameters increased by 1 SD mmHg were listed in Table 2. All the BP parameters (All stroke, IS, and HS) showed significant association with the risk of stroke incident at 0.001 level.

**Table 2:** Adjusted Hazard Ratios (HRs) of the four BP Parameters for stroke^a^

BP Parameters (per 1 SD mmHg increased)	All stroke		Ischemic Stroke		Hemorrhage stroke	
	HRs (95% CI)	*P*	HRs (95% CI)	*P*	HRs (95% CI)	*P*
SBP	1.751 (1.589–1.930)	< 0.001	1.619 (1.426–1.839)	< 0.001	2.008 (1.721–2.343)	< 0.001
DBP	1.377 (1.302–1.455)	< 0.001	1.316 (1.224–1.415)	< 0.001	1.488 (1.363–1.625)	< 0.001
PP	1.420 (1.299–1.552)	< 0.001	1.371 (1.224–1.537)	< 0.001	1.515 (1.311–1.750)	< 0.001
MAP	1.537 (1.426–1.656)	< 0.001	1.448 (1.313–1.596)	< 0.001	1.707 (1.517–1.922)	< 0.001

*BP* blood pressure, *SBP* systolic blood pressure, *DBP* diastolic blood pressure, *PP* pulse pressure, *MAP* mean arterial pressure, *BMI* body mass index, *HR* heart rate, *TC* total cholesterol, *TG* triglycerides, *HDL-C* high-density lipoprotein cholesterol, *LDL-C* low-density lipoprotein cholesterol

^a^ Included variables: age (years), sex, ethnicity, BMI, HR, current smoking, current drinking, diabetes mellitus, and anti-hypertension drug treatment, TC, TG, HDL-C, LDL-C

Table 3 demonstrates the adjusted HRs of BP parameters that had been standardized in the Cox proportional hazard model for stroke according to age categories. The value of HR for PP among hypertensive subjects aged less than 60 revealed slightly lower than that of SBP for all stroke incident [HR (95% CI): 1.635 (1.451–1.842) vs 1.734 (1.562–1.9)] and HS incident [HR (95% CI): 1.788 (1.490–2.145) vs 2.053 (1.757–2.398)]. However, the HR value of PP (1.531, 95% CI: 1.308–1.793, *P* < 0.001) was the highest among the four BP for IS events. The HR values of PP among hypertensive subjects aged over 60 showed the lowest for all stroke (1.228, 95% CI: 1.86–1.389, *P* = 0.001), IS (1.244, 95% CI: 1.066–1.451, *P* = 0.005), and HS (1.200, 95% CI: 0.972–1481, *P* = 0.090) events.

**Table 3:** Adjusted Hazard Ratios (HRs) of BP parameters for stroke according to age categories*

BP parameters	< 60 years (*n* = 3250)		≥ 60 years (*n* = 1847)	
	HRs (95% CI)	*P*	HRs (95% CI)	*P*
All stroke				
SBP	1.734 (1.562–1.924)	< 0.001	1.360 (1.216–1.522)	< 0.001
DBP	1.442 (1.288–1.616)	< 0.001	1.304 (1.150–1.479)	< 0.001
PP	1.635 (1.451–1.842)	< 0.001	1.228 (1.086–1.389)	0.001
MAP	1.696 (1.534–1.874)	< 0.001	1.337 (1.234–1.538)	< 0.001
Ischemic Stroke				
SBP	1.524 (1.322–1.756)	< 0.001	1.377 (1.198–1.582)	< 0.001
DBP	1.224 (1.041–1.439)	0.014	1.315 (1.125–1.537)	0.001
PP	1.531 (1.308–1.793)	< 0.001	1.244 (1.066–1.451)	0.005
MAP	1.482 (1.289–1.703)	< 0.001	1.392 (1.215–1.595)	< 0.001
Hemorrhage stroke				
SBP	2.053 (1.757–2.398)	< 0.001	1.328 (1.094–1.613)	0.004
DBP	1.694 (1.457–1.970)	< 0.001	1.280 (1.032–1.586)	0.024
PP	1.788 (1.490–2.145)	< 0.001	1.200 (0.972–1.481)	0.090
MAP	2.003 (1.730–2.320)	< 0.001	1.348 (1.113–1.632)	0.002

Using the Cox proportional hazards model to calculate the Hazard Ratios of four BP parameters that was standardized for the risk of stroke incident. Adjusted variables: age (years), sex, ethnicity, BMI, HR, current smoking, current drinking, diabetes mellitus, anti-hypertension drug treatment, TC, TG, HDL-C, LDL-C

*BP* blood pressure, *SBP* systolic blood pressure, *DBP* diastolic blood pressure, *PP* pulse pressure, *MAP* mean arterial pressure, *BMI* body mass index, *HR* heart rate, *TC* total cholesterol, *TG* triglycerides, *HDL-C* high-density lipoprotein cholesterol, *LDL-C* low-density lipoprotein cholesterol

Table 4 summarizes the results of the predictive power between the four BP parameters for all stroke, IS, and HS. For all stroke, SBP indicated the largest coefficient and PP was lower than SBP, and the difference between SBP and PP were statistically significant (*β*_SBP_ = 0.435 > *β*_MAP_ = 0.430 > *β*_PP_ = 0.351 > *β*_DBP_ = 0.322). Although the coefficient of MAP was lower than SBP marginally, the difference between MAP and SBP were not statistically significant (*P* = 0.756). Identical results were observed in the IS and HS events.

**Table 4:** Comparisons of a Predictive Power between the four BP Parameters for stroke

BP parameters	*β* coefficient	*P*
All stroke		
SBP vs DBP	0.435 vs 0.322	< 0.001
SBP vs PP	0.435 vs 0.351	< 0.001
SBP vs MAP	0.435 vs 0.430	0.756
DBP vs PP	0.322 vs 0.351	0.099
DBP vs MAP	0.322 vs 0.430	< 0.001
PP vs MAP	0.351 vs 0.430	< 0.001
Ischemic Stroke		
SBP vs DBP	0.378 vs 0.255	< 0.001
SBP vs PP	0.378 vs 0.316	< 0.001
SBP vs MAP	0.378 vs 0.370	0.639
DBP vs PP	0.255 vs 0.316	< 0.001
DBP vs MAP	0.255 vs 0.370	< 0.001
PP vs MAP	0.316 vs 0.370	0.002
Hemorrhage stroke		
SBP vs DBP	0.539 vs0.425	< 0.001
SBP vs PP	0.539 vs 0.415	< 0.001
SBP vs MAP	0.539 vs 0.535	0.777
DBP vs PP	0.425 vs 0.415	0.540
DBP vs MAP	0.425 vs 0.535	< 0.001
PP vs MAP	0.415 vs 0.535	< 0.001

Using the Cox proportional hazards model to calculate the *β*-coefficient of four BP parameters that was standardized for the risk of stroke incident. Adjusted variables: age (years), sex, ethnicity, BMI, HR, current smoking, current drinking, diabetes mellitus, and anti-hypertension drug treatment, TC, TG, HDL-C, LDL-C

*BP* blood pressure, *SBP* systolic blood pressure, *DBP* diastolic blood pressure, *PP* pulse pressure, *MAP* mean arterial pressure, *BMI* body mass index, *HR* heart rate, *TC* total cholesterol, *TG* triglycerides, *HDL-C* high-density lipoprotein cholesterol, *LDL-C* low-density lipoprotein cholesterol

## Discussion

The present study provided an in-depth analysis of associations between blood pressure parameters and stroke among rural Chinese persons with hypertension. The results indicated that all the BP parameters were associated with the outcomes, significantly. The study results revealed that SBP was a more effective predictor of the outcomes than DBP and PP excluding MAP.

The increase of PP enhances arteries to bear more stress, which leads to the increase of elastic component fatigue and fracture rate and weaken the lining of blood vessels. Current studies have shown that PP’s ability to predict adverse cardiovascular events were controversial [7, 8, 11–21]. Framingham Heart Study had indicated that PP was still a predictor for stroke incident after adjusted by SBP and DBP, even though its predictive ability was inferior to SBP and DBP. Another study had showed that PP was a powerful predictor of stroke incident when DBP and MAP were adjusted, but not when SBP was adjusted [13]. The present study (based on hypertensive patients) showed that the four BP parameters were independent risk factors for stroke after adjusting some acknowledged influential factors of stroke. However, when SBP and DBP were incorporated into the original regulatory factors, the effect of MAP and PP disappeared. The present results doesn’t agree with previous study that PP was an independent risk predictor of stroke in the general population [8].

To eliminate the impact of multiple collinearities among the four BP parameters, the four BP parameters were incorporated into the model to calculate the corresponding coefficient difference. For stroke and its two subtypes (IS and HS), SBP obtained the highest HRs value, which was followed by MAP while the HRs value of PP was lower than the above two and higher than DBP. The comparison results of stroke prediction capacity showed that the prediction ability of SBP was significantly higher than that of DBP and PP, and no significant differences were observed with MAP. The above results suggested that MAP was a sensitive indicator of onset stroke events in hypertensive patients.

It has been observed that BP (DBP and SBP) increasing in a parallel manner until age ≤ 60, as age over 60 years, SBP continues to rise, but DBP begins to decrease as a result of reducing revers capacity of aorta with advancing age [22, 27]. Therefore, PP may be regarded as a powerful indicators of BP for the elderly [8]. The Framingham heart study indicated that the risk of CHD increased by 23% when PP increasing in every 10 mmHg [13]. This association between CHD risk and PP was observed in patients over 50 years old, especially those over 60 years old [14]. Therefore, the age stratification analysis was carried out in our study with the cut-off value of 60 years old (Table 3). The current study result could not verify that PP was more applicable to the elderly population for stroke incident, however, the outcome revealed a high predictive power for the relatively low age group in hypertensive patients.

The main adverse manifestations of stroke are IS and HS, and their incidence increasing with age [28]. As people’s life expectancy increases, the burden of stroke on people and society worldwide will become heavier. 75% of stroke patients were measured to had a high BP at the time of admission [29]. Hypertension-related stroke is a pervasive, on the other hand its preventable public health related problem. Hypertension has been shown to be the most common and the most influential risk factor for IS and HS, and the incidence of IS and HS could be significantly reduced by effective hypertensive therapy [30, 31]. It is particularly crucial to control BP and to use sensitive BP parameters. Both PP and MAP were calculated from the main components of BP (SBP and DBP). PP mainly reflects two aspects of ventricular ejection volume and wave reflection [13, 32]. In addition to reflecting ventricular ejection and peripheral vascular resistance, MAP was considered as the main determinant of cerebral blood flow [13, 32], which was critical for brain tissue damage [33]. This may be the reason that MAP could predict stroke better than PP and this finding corporate the previous result [34].

### Limitation

Some critical limitations need to be considered. Firstly, the present cohort study was composed of adults who were completely from rural areas in China, and their diversity may be limited. Secondly, in this study only baseline BP values was used, it should be taken into account that stroke vascular damage is a dynamic and complex process over time. Further study are required to the relationship between dynamic BP parameters levels and stroke and to clarify its pathogenesis.

## Conclusion

Within the limitation of the current study, the following conclusion can be drawn. The SBP was supported as the main indicator of BP parameters in predicting stroke risk in hypertensive patients. Although PP could predict stroke risk in hypertensive patients, its predictive ability was lower than SBP. However, the assessment and management of MAP could be considered in clinical BP control and reducing the risk of stroke.

## Data Availability

The datasets used and/or analyzed during the current study are available from the corresponding author on reasonable request.

## Abbreviations

BMI: Body mass index
BP: Blood pressure
CAD: Concluded angina pectoris, myocardial infarction, arrhythmia
CHD: Coronary heart disease
CI: Confidence interval
CVD: Cardiovascular disease
DBP: Diastolic BP
DM: Diabetes mellitus
HDL-C: High-density lipoprotein cholesterol
HR: Hazard ratio
HS: Hemorrhage stroke
ICH: Intracerebral hemorrhage stroke
IS: Ischemic stroke
LDL-C: Low-density lipoprotein cholesterol
MAP: Mean arterial pressure
MONICA: Multinational monitoring of trends and determinants in cardiovascular disease
PP: Pulse pressure
SAH: Subarachnoid hemorrhage stroke
SBP: Systolic BP
SD: Standard deviations
TC: Total cholesterol;
TG: Triglycerides
TIA: Transient ischemic attacks
